# Willingness to Pay (WTP) for COVID-19 Vaccine Booster Dose and Its Determinants in Indonesia

**DOI:** 10.3390/idr14060101

**Published:** 2022-12-11

**Authors:** Harapan Harapan, Malik Sallam, Raisha Fathima, Hendrix Indra Kusuma, Samsul Anwar, Widhy Yudistira Nalapraya, Adityo Wibowo, Ketut Dewi Kumara Wati, Ayunda Medina, Anna Hanifa Defrita, Yesi Astri, Arie Prasetyowati, Nurfarahin Nurfarahin, Afriyani Khusna, Setya Oktariana, Sarifuddin Anwar, Milza Oka Yussar, Siti Khotimah, Bahagia Willibrordus Maria Nainggolan, Putri Rizki Amalia Badri, Raden Argarini, Wira Winardi, Khan Sharun, Rosaria Indah, Yogambigai Rajamoorthy, Abram L. Wagner, Mudatsir Mudatsir

**Affiliations:** 1 Medical Research Unit, School of Medicine, Universitas Syiah Kuala, Banda Aceh 23111, Indonesia; 2 Tropical Disease Centre, School of Medicine, Universitas Syiah Kuala, Banda Aceh 23111, Indonesia; 3 Department of Microbiology, School of Medicine, Universitas Syiah Kuala, Banda Aceh 23111, Indonesia; 4 Tsunami and Disaster Mitigation Research Center (TDMRC), Universitas Syiah Kuala, Banda Aceh 23111, Indonesia; 5 Department of Pathology, Microbiology and Forensic Medicine, School of Medicine, The University of Jordan, Amman 11942, Jordan; 6 Department of Clinical Laboratories and Forensic Medicine, Jordan University Hospital, Amman 11942, Jordan; 7 Department of Translational Medicine, Faculty of Medicine, Lund University, 22184 Malmö, Sweden; 8 Department of Biology, Faculty of Mathematics and Natural Sciences, Universitas Syiah Kuala, Banda Aceh 23111, Indonesia; 9 Biology Education Department, Faculty of Tarbiyah and Teacher Training, Universitas Islam Negeri Ar-Raniry, Banda Aceh 23111, Indonesia; 10 Department of Statistics, Faculty of Mathematics and Natural Sciences, Universitas Syiah Kuala, Banda Aceh 23111, Indonesia; 11 Pulmonology and Respiratory Medicine, Medical Faculty of Universitas Islam Bandung, Bandung 40116, Indonesia; 12 Department of Pulmonology and Respiratory Medicine, Faculty of Medicine, Universitas Lampung, Bandar Lampung 35145, Indonesia; 13 Department of Child Health, Faculty of Medicine, Universitas Udayana, Denpasar 80234, Indonesia; 14 Faculty of Medicine, Universitas Jambi, Jambi 36373, Indonesia; 15 Neurology Department, Faculty of Medicine, Universitas Muhammadiyah Palembang, Palembang 30263, Indonesia; 16 Mungkid Community Health Center, Magelang 56512, Indonesia; 17 Department of Pulmonology and Respiratory Medicine, Faculty of Medicine, Tadulako University, Palu 94148, Indonesia; 18 Public Health Faculty, Universitas Muhammadiyah Aceh, Banda Aceh 23245, Indonesia; 19 Biochemistry Laboratory, Medical Faculty of Mulawarman University, Samarinda 75119, Indonesia; 20 Undergraduate Program in Medicine, Faculty of Medicine, Universitas Sumatera Utara, Medan 20155, Indonesia; 21 Public Health Department, Medical Faculty of Universitas Muhammadiyah Palembang, Palembang 30263, Indonesia; 22 Department of Medical Physiology and Biochemistry, Universitas Airlangga, Surabaya 60132, Indonesia; 23 Department of Pulmonology and Respiratory Medicine, School of Medicine, Universitas Syiah Kuala, Banda Aceh 23111, Indonesia; 24 Division of Surgery, ICAR-Indian Veterinary Research Institute, Izatnagar, Bareilly 243122, India; 25 Medical Education Unit, School of Medicine, Universitas Syiah Kuala, Banda Aceh 23111, Indonesia; 26 Department of Economics, Faculty of Accountancy and Management, Universiti Tunku Abdul Rahman, Kajang 43000, Malaysia; 27 Department of Epidemiology, School of Public Health, University of Michigan, Ann Arbor, MI 48109, USA

**Keywords:** willingness-to-pay, COVID-19, booster dose, vaccine, vaccination, Indonesia

## Abstract

Willingness to pay (WTP) for booster doses of coronavirus disease 2019 (COVID-19) vaccines is an under studied research topic. Therefore, the current study aimed to investigate the WTP for the booster doses of COVID-19 vaccines and its predictors in Indonesia using an online survey distributed all over the provinces of this low-middle-income country. The WTP was evaluated using a basic dichotomous contingent valuation approach, and its associated determinants were evaluated using a linear regression model. Out of 2935 responders, 66.2% (1942/2935) were willing to pay for a booster dose of the COVID-19 vaccine. The majority of respondents (63.5%) were willing to pay within a price range of 100,000–500,000 Indonesian rupiah (IDR), i.e., USD 6.71–33.57. Being older than 40 years, having a higher educational level, having a higher income, knowing and understanding that booster doses were important, and having a vaccine status that is certified *halal* (permissible in Islamic law), were all associated with a higher WTP for the booster dose of COVID-19 vaccines. The study findings imply that the WTP for a booster dose of COVID-19 vaccination in Indonesia is lower compared to acceptance of vaccines provided free of charge. This WTP data can be utilized to develop a pricing scheme for the booster doses of COVID-19 vaccination in the country with potential benefits in other low-income countries. The government may be required to provide subsidies for the herd immunity vaccination process to proceed as anticipated. Furthermore, the public community must be educated on the importance of vaccination as well as the fact that the COVID-19 epidemic is far from being over.

## 1. Introduction

The emergence of new variants of the severe acute respiratory syndrome coronavirus 2 (SARS-CoV-2) with higher transmissibility necessitates booster dose vaccination for the global community [1,2,3]. Another factor that highlights the need for booster dose vaccination is the short-lived protective immunity following the primary dose COVID-19 vaccination [4,5], indicating that booster doses of COVID-19 vaccination are critical for proper control of the ongoing pandemic [6]. The concept of booster dose vaccination involves the repeated administration of the vaccine to boost immunity [7]. Based on a systematic review study, COVID-19 vaccines provide significant protection against the infection, but their effectiveness wane over time [8]. Specifically, immunity against COVID-19 infection decreased from 83% after the first month to 22% following five or more months of full primary vaccination [8]. Consequently, booster doses of COVID-19 vaccination appear warranted over the long term [9]. However, the cost of vaccines can be a barrier to self-pay vaccine acceptance [10]; hence, assessing the willingness to pay (WTP) for vaccination is necessary for vaccine policy making and promoting booster vaccination acceptance, particularly in low-middle income countries such as Indonesia.

In the context of healthcare, WTP is a method for determining how much people are willing to spend on healthcare plans, services, and medical interventions [11,12]. Originally derived from the economic literature, WTP was later adopted in various disciplines, particularly to assess judgment in making decisions [13], in this case, the decision to receive a booster dose vaccine or not. Booster dose vaccine WTP research is still scarce and under studied; as far as we know, research on booster dose WTP has only been initiated in China [11,14,15]. Although these studies found that booster dose vaccines are widely accepted in China (nearly 85%), the WTP was generally low, with individuals willing to pay less than 200 Chinese Yuan Renminbi (CNY), i.e., around USD 28.49, and refusing to pay more [11]. Another survey study found that only 14.5% (155/1072) of healthcare workers (HCWs) were willing to pay 100 CNY (equivalent to USD 14.25) for a booster vaccine, while 53% were unwilling to pay [14]. Sociodemographic, individual attitudes, and beliefs are factors that greatly affect booster dose vaccine acceptance and the WTP for booster doses [11,16,17]. Being a HCW, having higher incomes and having a high risk of COVID-19 were previously identified as factors associated with higher WTP for the vaccine [17].

The Indonesian government has previously provided all COVID-19 vaccines free of charge for the residents in this southeast lower-middle income Asian country. However, due to declining immunity and continuous emergence of SARS-CoV-2 variants, it may be necessary to re-vaccinate in the future. Vaccines are likely to be expensive given the limited medical resources available. As a result, determining the public’s willingness to pay for the COVID-19 vaccine to achieve proper prevention and control is critical. The purpose of this study was to determine whether Indonesians are willing to pay for a booster dose of the COVID-19 vaccine and the factors associated with it.

## 2. Methods

### 2.1. Study Design and Eligible Criteria

By enlisting 31 collaborators who represented the Indonesia’s five large islands, a cross-sectional online survey was distributed to every province in the country. The inclusion criteria for participation were: (1) being an Indonesian citizen ≥ 18 years old, and (2) having internet access. Those without internet access were unable to participate in this study. 

The protocol was reviewed and approved by the ethical committee of the Faculty of Medicine at Universitas Syiah Kuala (Approval number: 008/EA/FK/2022). All procedures were carried out in compliance with the principles of the Declaration of Helsinki. Participants were provided with an electronic informed consent form which was mandatory before completing the questionnaire, and their information was kept anonymous.

### 2.2. Questionnaire Structure

The questionnaire consisted of several sections: (1) basic demographic information (age, gender, marital status, the highest attained education, religion, and monthly income); (2) knowledge, awareness, acceptance, perception, the perceived severity, the perceived benefit, and the perceived barriers on getting the booster dose COVID-19 vaccine, and (3) WTP for the booster dose COVID-19 vaccines. The WTP questions were adopted from previous study with modifications [18].

WTP is the highest amount of money a person would likely pay for vaccination [17]. To measure the WTP, a double-bounded dichotomous choice technique was used as commonly used in COVID-19 and non-COVID-19 vaccines previously [19,20,21,22]. The respondents were provided the scenario and question “If the effectiveness of the booster dose of the COVID-19 vaccine is 95% and there is a 5% chance of side effects such as fever or local pain, will you pay 250,000 Indonesian rupiah (IDR) for the vaccine?” Then, they were given a “yes” or “no” choice. Those who choose “yes” the bid was increased to 500,000 IDR, one million IDR, or two million IDR. Meanwhile, those who answer “no” to the first price, the bid was reduced to 125,000 IDR, 60,000 IDR, 30,000 IDR, and 15,000 IDR. Those who were unwilling to pay the 15,000 IDR were given the option of determining the lowest price they are willing to pay. For each respondent, the highest amount of money that they were willing to spend to purchase the vaccine was defined as WTP. The WTP was then converted to USD using an IDR-to-USD exchange rate of USD 1 equivalent to IDR 14,835, i.e., the 31 August 2022 currency exchange rate.

### 2.3. Procedure

The data were collected between 1 and 15 August 2022, utilizing an electronic link distributed via the collaborators’ social network. The platforms used in questionnaire distribution were WhatsApp, Telegram, Messenger, Line, Facebook, Instagram, and Twitter. Before completing the questionnaire voluntarily, informed consent was supplied by the respondents. By not gathering any identifiable details of personal information, anonymity and confidentiality were preserved.

### 2.4. Data Analysis

All analyses were carried out using IBM SPSS Statistics for Windows (Armonk, NY, USA: IBM Corp). For continuous variables, descriptive statistics were reported in means and standard deviations (mean ± SD), whereas categorical variables were summarized using frequency and percentage. A linear regression model was utilized to evaluate the factors associated with WTP. This method has been commonly used previously [17,23,24,25]. Prior to analysis using the model, diagnostic assessments were conducted to ensure that the assumptions of the model were fulfilled (i.e., multicollinearity, heteroscedasticity, and residual normality). To ensure the data met all the assumptions of the linear regression model, the data were transformed using a natural logarithm function. This step was taken because the data violated multicollinearity, heteroscedasticity and residual normality when tested using the variance inflation factor (VIF) [26], Glejser test [27] and Kolmogorov-Smirnov test [28], respectively. The log-transformed WTP data were on a ratio scale. In the initial model, all determinants were included and all determinants with *p* < 0.05 in the model were entered in the final model. For each variable, one of the categories was used as reference category (*R*). 

To calculate the mean of estimated WTP, Exp(Xβ^+σ^2/2) was used, in which β^ is estimated regression coefficient and σ^2 is the mean squared error (MSE) of the model [29,30]. The 95% confidence interval (CI) of the mean of estimated WTP was calculated. Statistical significance was defined as a *p* value < 0.050.

## 3. Results

### 3.1. Sociodemographic and Characteristics of Respondents

Out of 2935 people who filled out the survey, 66.2% (1942/2935) of the respondents were willing to pay for a booster dose of the COVID-19 vaccine and 33.8% (993/2935) were unwilling to pay (Table 1). The majority of respondents were willing to pay within a price range of 100,000–500.000 IDR, followed by <100,000 IDR (63.5% and 28.4%, respectively). 

Almost 70% of respondents willing to pay were women, aged 21–30 years in age (49.2%), single (57.2%), and graduated from university (66.1%). Respondents were dominated by Muslims (78.3%). Almost half of the respondents were employed for wages (49.7%) with a monthly income under 3 million IDR (56.1%) (Table 2).

Of the respondents, 26.8% had a family member that was seriously ill or died as a result of COVID-19, had been infected with COVID-19 (49.3%), received the first and second dose of the vaccine, and been infected again after vaccination (30.8%). A variable percentage of the study respondents believed that vaccines could stimulate and improve the immune system, lower the hospitalization rate, and protect themselves, their families, and the people around them (Table 3). The factors that influence the WTP for booster dose of COVID-19 vaccination in Indonesia are also presented in (Table 3).

### 3.2. Factors Associated with WTP for the Booster Dose of the COVID-19 Vaccine

The unadjusted initial linear regression model revealed that 16 of the 35 variables had at least one category with a *p* value < 0.050. The final linear regression model included all of these explanatory factors. The results of the multivariate model analysis indicated that age, education level, religion, monthly income, having been vaccinated against influenza in the last 5 years, the knowledge that booster dose vaccines can increase immunity, and awareness that booster dose vaccination can protect people who cannot be vaccinated, perception of complications after booster dose vaccine, perceived benefit, and halal status of the vaccine, were all associated with WTP (Table 4).

Among participants who were willing to pay, respondents over 41–50 years had a higher WTP of approximately USD 1.47 than those around 31–40 years, and undergraduate/postgraduate respondents had a higher WTP compared to elementary or high school graduates, of about USD 2.62. Respondents who identified themselves as Catholic and other religions (Hindu/Buddhist/Atheist or agnostic/Confucian) had greater WTPs than Muslims, of around USD 2.26 and USD 2.46, respectively. Those who worked for wages, were students, or retired, had a higher WTP of approximately USD 2.56 and USD 2.62 compared to those who worked as homemakers or were self-employed.

Respondents with a monthly income of more than 10 million IDR were willing to pay around USD 3.02 higher than those with an income of less than 3 million IDR. Respondents who had received an influenza vaccination in the last five years had a greater WTP than those who had not, which was roughly USD 2.32. Those who were not aware that the booster dosage can improve immunity after the second dose and that the booster dose can protect unvaccinated people had greater WTP than those who had such knowledge (approximately USD 1.39 and USD 1.57, respectively). Compared to those who disagreed with the term “I believe that natural immunity is sufficient, and I do not need to be vaccinated”, respondents who neither agree nor disagree had a higher WTP of approximately USD 1.44. Those who agreed that “COVID-19 infection is harmless, so I do not have to be vaccinated” had a higher WTP approximately USD 2.50 compared to those who disagreed. Meanwhile, respondents who answered neither agree nor disagree with the statement “Complications may arise after receiving the booster dose” had a higher WTP of around USD 1.64 compared to respondents who answered disagree. Respondents who agreed and neither agreed nor disagreed that getting vaccinated would minimize their risk of contracting infection or infecting others had greater WTP than those who disagreed (about USD 3.41 and USD 3.65, respectively). Respondents who were concerned about the halal status of the COVID-19 booster dose had a higher WTP than those who were not concerned (USD 1.55).

## 4. Discussion

The major finding of the current study was the demonstration of much lower intention to receive the booster doses of COVID-19 vaccination if payment is needed. A recent study among the general public in Indonesia showed that the willingness to receive the booster doses of COVID-19 vaccines, if provided free of charge, was 95% [31]. The current study with the same target population showed that the need to pay for the vaccine was linked with a discernable decline of willingness to get the booster doses. Specifically, the WTP for a booster dose was only 66% in the current study sample. This result was expected given the previous evidence that affordability of vaccines is an important driving factor of vaccination convenience. The importance of vaccination convenience is manifested by its inclusion in various models conceived to understand and measure the predictors of vaccination hesitancy (e.g., 3C, 5C and 7C models) [32,33,34]. Therefore, paying for vaccines in general, including the booster doses of COVID-19 vaccines, can be a major hurdle to the efforts aimed to promote vaccination. Consistent with this observation, several previous studies showed a similar lower rate of vaccine acceptance if payment was needed in the context of COVID-19 [18,35], HPV [36], and influenza vaccination [37].

The relevance of the current study is that, first, the sustainability of vaccine procurement can be compromised over time particularly in low-income countries [38]. Second, there is growing evidence showing the value of booster doses of COVID-19 vaccines stemming from the continuous emergence of SARS-CoV-2 variants with immune escape potential, and the declining immunity following the uptake of the primary vaccine doses [5,39,40,41,42]. Third, the continuous need to receive the booster doses for proper control of COVID-19 might lead to vaccination fatigue, which highlights the need for separate studies to evaluate the general public attitude towards repeated vaccination [43]. The phenomenon of vaccination fatigue is associated with large infectious disease outbreaks, and it entails inaction towards vaccine information and recommendation [43]. Consequently, this can lead to high perceived burden of such information with burnout and hesitancy to receive the booster doses, let alone paying for these doses. Therefore, the intention to get the booster doses of COVID-19 vaccines might be compromised, particularly if payment is needed. The current study was conducted in Indonesia which is a low-income country in southeast Asia. Previous studies showed that the rates of COVID-19 vaccine acceptance in Indonesia were among the highest in the world [44,45,46]. However, the economic burden of the pandemic in a low-income setting can force governments to require payment for vaccination [47], with subsequent risk of hesitancy to receive the booster doses of COVID-19 vaccines. 

In this study, WTP for the booster dose of COVID-19 vaccines in Indonesia tended to be relatively low at 66.2% (1942/2935), yet it is not definite that if this booster dose vaccine is provided free of charge, the public will refuse it, as evidenced by our previous study on the acceptance of booster COVID-19 vaccination in Indonesia [31]. Variability in WTP for the primary COVID-19 vaccination has been reported in different studies globally [17,48,49,50,51,52,53]; however, our study can be considered among the first studies to assess WTP for the booster dose of the vaccine. A recent study that was conducted among HCWs in China showed that the WTP for the booster dose was reported among only 47% of the participants [14].

In this study, the majority of Indonesians (63.5%) were willing to pay 100,000–500,000 IDR (equivalent to USD 6.71–33.57) for the booster dose vaccination. In the previous study that assessed WTP for the primary COVID-19 vaccination in Indonesia [17], 1065/1359 Indonesians (78.3%) were showed WTP for the vaccine, and most respondents were willing to pay USD 30.94 (mean: USD 57.20) for the primary COVID-19 vaccination [17]. 

Many factors influence a person’s WTP level for the COVID-19 vaccine, as previously reported [17,48,49,50,51,52,53]. In the current study, WTP was associated with age, education level, religion, monthly income, previous uptake of influenza vaccines in the last 5 years, the knowledge that booster dose vaccines can increase immunity, and awareness that booster dose vaccination can protect people who cannot be vaccinated, perception of complications after booster dose vaccine, perceived benefit, and halal status of the vaccine.

Individuals aged between 41–50, highly educated and earning more than 10 million IDR were willing to spend more for booster doses of COVID-19 vaccines. As mentioned earlier, WTP is influenced by factors such as age, education level, and monthly income, according to numerous studies [14,17,50,54]. More knowledge of the danger of COVID-19 can be ascribed to age and education, with lower levels of complacency linked to higher acceptance of vaccination [16]. According to Cerda et al. [50], individuals with lower levels of education showed less willingness to get vaccinated. This was linked to low health literacy and being less aware of threats associated with the disease [55,56]. Furthermore, in this study, individuals older than 40 years showed higher WTP, which can be linked to higher perceived threats due to previous evidence showing that older individuals are at higher risk of developing severe disease with higher risk of mortality [57,58]. Thus, it is understandable to observe higher willing to receive the vaccine among individuals who are aware that COVID-19 can be a serious disease [54,59], with subsequent higher willingness to pay for the vaccine even if it is slightly more expensive. A higher WTP tendency increase was also previously linked with higher monthly income [17,50]. Previous studies have shown that income is positively correlated with COVID-19 vaccine WTP, which is related to an individual’s ability to pay [17,18,50]. Therefore, if payment for the booster dose is required, it is necessary to consider providing these doses at an affordable price. In the context of the current study results, if the vaccines provided are relatively expensive, the WTP would decline to a large extent, with individuals having an income of 3 million being unwilling to be vaccinated, which is translated into more than half of the Indonesian population. This will have an impact on the government’s goal of vaccinating 70% of the population in order to achieve herd immunity [60].

In this study, we also found that people who reported uptake of influenza vaccines in the last 5 years showed a higher WTP for the booster COVID-19 vaccination. This result can be linked with higher likelihood of positive perceived benefits associated with a previous history of vaccination. Getting the COVID-19 vaccine is a safer and more reliable way to build protection rather than having natural immunity a result of the disease. COVID-19 vaccination helps to protect individuals by creating an antibody response without having to experience a potentially severe illness or post-COVID conditions [61]. In addition, COVID-19 vaccination lowers the rate of hospitalization care and the risk of death [62,63,64]. However, studies show that the level of protection of vaccination against infection decreases over time (after 6 months) [5,65]. In addition, new variants of the SARS-CoV-2 virus continue to emerge with vaccine resistance capabilities [42,66], so a subsequent booster dose is needed to retain immunity [5]. Interestingly, the knowledge that booster dose vaccines can increase immunity, and awareness that booster dose vaccination can protect people who cannot be vaccinated, increases WTP in those without this knowledge compared to those with it.

In addition to the factors indicated above, perception of complications after booster dose vaccine was known to be associated with WTP for booster doses. Individuals who believed that booster doses of vaccines are less safe or who were concerned about the possibility of adverse event occurrence following vaccination had a low WTP, whereas those who believed that vaccination was safe were more willing to pay a higher price. Vaccination hesitancy related to vaccine security was experienced not only by common citizens, but also by health students and those who work in the health sector, as has been widely reported in previous studies, both in developing countries such as Sudan [67], Uganda [68], India [69], Turkey [70], and in developed countries as well, such as the United States [71,72], Poland [73], Slovenia [74] and China [75]. The main reason for their skepticism was that they had heard negative information concerning the COVID-19 vaccine [67,68].

Indonesian society, which is predominantly Islamic, pays great attention to the “halalness” of a product following Sharia Law. Muslims are obligated to follow Sharia Law, which is authoritative in Islam [76]. Many individuals were still skeptical about vaccines with the fear that such vaccines may contain substances prohibited in Islam. Substances used in vaccine manufacturing may be of animal origin, including swine or derivatives, dead animals, or blood, all of which are *haram* or forbidden for Muslims [77,78]. In Muslim-populated countries, *halal* certification administrators award the *halal* certificate to applicants based on the Holy Quran. Meanwhile, many COVID-19 vaccines do not have these certificates. As a result, they rely on ulama *fatwas* (opinions or interpretations on an issue related to Islamic law by the Indonesian Ulama Council (MUI)). A study on the Islamic sharia perspective on COVID-19 vaccines detailed the legal provisions of various forms of vaccinations used in Indonesia [79]. The MUI declared the Sinovac and Anhui vaccines *halal* since they were manufactured without the usage of porcine derivatives [77,80]. MUI asserts AstraZeneca is *haram*-permittable; it is *haram* since it employs porcine trypsin in the early stages of manufacturing, but it is permissible to use (or *Mubah*) due to the urgency of controlling COVID-19 [81]. In the meantime, several additional vaccinations, including Moderna, J&J, Sputnik V, and CanSino, were not *halal/haram* certified [79].

This research has certain limitations as follows. First, in order to observe the impact of *halal* status on acceptance and WTP for the booster dose of COVID-19 vaccines, it would have been preferable if the religion included was exclusive of Islam only, as it is highly forbidden in Islam to use something that contains forbidden ingredients. Second, because this study used an internet-based platform, individuals who did not use gadgets or have internet access were unable to participate, and this might cause selection bias. Third, the comparisons made with the previous studies on WTP for the booster dose of COVID-19 vaccines should be done with caution considering the variability in survey instruments used for this aim. An important caveat of the WTP assessment, according to Tung et al., is that it is hypothetical [11]. Regardless of the individual’s response to the survey instrument, the individual had not paid the actual price by the end of the study. What people claim they will do, and what they actually do, may be inconsistent [11,82].

## 5. Conclusions

According to our findings, the WTP of the COVID-19 booster dose in Indonesia is highly influenced by age, education level, monthly income, understanding of the benefits and public awareness of vaccination, and the vaccine’s *halal* status. The government appears to need to offer subsidies considering the discernible decline in vaccine acceptance if payment is needed, so that the herd immunity vaccination process may go as planned. Furthermore, the public must be educated on the need for vaccination as well as the fact that the COVID-19 pandemic is not over.

## Figures and Tables

**Table 1:** Willingness to pay and the prices the participants were willing to pay for a booster dose of COVID-19 vaccine (*n* = 2935).

Characteristic	Number	Percentage
Willing to pay (*n* = 2935)		
Yes	1942	66.2
No	993	33.8
Price that respondent willing to pay (*n* = 1942)		
Less than 100,000 IDR ^1^	552	28.4
100,000–500,000 IDR	1234	63.5
501,000–1,000,000 IDR	70	3.6
1,001,000–2,000,000 IDR	86	4.4

^1^ IDR: Indonesian Rupiah.

**Table 2:** Demographic characteristics of the participants who were willing to pay for a booster dose COVID-19 vaccine in Indonesia (*n* = 1942).

Characteristic	Number	Percentage
Sex		
Male	590	30.4
Female	1352	69.6
Age		
≤20	295	15.2
21–30	956	49.2
31–40	464	23.9
41–50	128	6.6
51–60	71	3.7
>60	28	1.4
Marital status		
Single	1111	57.2
Married	796	41
Divorced/widow/widower	35	1.8
The highest educational level		
Elementary/high school	429	22.1
Diploma	1284	66.1
Undergraduate/graduated	229	11.8
Religion		
Islam	1521	78.3
Christian (Protestant)	166	8.5
Catholic	123	6.3
Other (Hindu/Buddha/Atheism or agnosticism/Confucianism)	132	6.8
Occupation		
Self-employed	24	1.2
Employed for wages	966	49.7
Homemaker	63	3.2
Student/Retired/unable to work/others	889	45.8
Monthly household income (IDR) ^1^		
<3 million	1090	56.1
3–5 million	256	13.2
5–10 million	363	18.7
>10 million	233	12

^1^ IDR: Indonesian Rupiah.

**Table 3:** Factors of WTP of booster dose of COVID-19 vaccine in Indonesia (*n* = 1942).

Characteristic	Number	Percentage
Having family member seriously ill or died caused by COVID-19?		
Yes	520	26.8
No	1422	73.2
Having received influenza vaccination for the last 5 years?		
Yes	360	18.5
No	1582	81.5
Have you ever been infected with COVID-19?		
Yes	958	49.3
No	984	50.7
Type of COVID-19 vaccine received for the 1st dose		
Sinovac	1680	86.5
AstraZeneca/Moderna/Pfizer/Sinopharm/others	262	13.5
Type of COVID-19 vaccine received for the 2nd dose		
Sinovac	1594	82.1
AstraZeneca/Moderna/Pfizer/Sinopharm/others	348	17.9
Have you ever been infected with COVID-19 after getting vaccinated?		
Yes	599	30.8
No/I do not know	1343	69.2
Booster dose can provide immune better after the second dose		
Yes	1695	87.3
No/I do not know	247	12.7
Booster dose can stimulate antibody production to fight COVID-19 infection		
Yes	1800	92.7
No/Not sure	142	7.3
Booster dose can lower hospitalization rate if infected by COVID-19		
Yes	1760	90.6
No/Not sure	182	9.4
Booster dose can protect the unvaccinated people		
Yes	1478	76.1
No/Not sure	464	23.9
The COVID-19 pandemic has greatly affected my source of income		
Agree or strongly agree	1433	73.8
Neither agree nor disagree	438	22.6
Disagree or strongly disagree	71	3.7
The COVID-19 pandemic has greatly affected my social life		
Agree or strongly agree	1731	89.1
Neither agree nor disagree	186	9.6
Disagree or strongly disagree	25	1.3
The booster dose is important to protect the public from COVID-19		
Yes	1846	95.1
No/I do not know	96	4.9
I believe that natural immunity is sufficient and I do not need to be vaccinated		
Agree or strongly agree	208	10.7
Neither agree nor disagree	295	15.2
Disagree or strongly disagree	1439	74.1
COVID-19 infection is harmless, so I do not have to be vaccinated		
Agree or strongly agree	151	7.8
Neither agree nor disagree	113	5.8
Disagree or strongly disagree	1678	86.4
I am not sure vaccination is effective against COVID-19		
Agree or strongly agree	245	12.6
Neither agree nor disagree	302	15.6
Disagree or strongly disagree	1395	71.8
I am worried about any adverse side effects or allergic reactions when vaccinated with booster doses		
Agree or strongly agree	1003	51.6
Neither agree nor disagree	648	33.4
Disagree or strongly disagree	291	15
I believe a booster dose COVID-19 vaccine is very important		
Agree or strongly agree	1667	85.8
Neither agree nor disagree	268	13.8
Disagree or strongly disagree	7	0.4
Booster dose is useful for protecting people from COVID-19		
Agree or strongly agree	1738	89.5
Neither agree nor disagree	193	9.9
Disagree or strongly disagree	11	0.6
Booster dose is safe		
Agree or strongly agree	1640	84.4
Neither agree nor disagree	290	14.9
Disagree or strongly disagree	12	0.6
Complications may arise after receiving the booster dose		
Agree or strongly agree	237	12.2
Neither agree nor disagree	727	37.4
Disagree or strongly disagree	978	50.4
I am worried about the unexpected effect of booster dose in the future		
Agree or strongly agree	698	35.9
Neither agree nor disagree	734	37.8
Disagree or strongly disagree	510	26.3
I believe the booster dose has good effectiveness		
Agree or strongly agree	1662	85.6
Neither agree nor disagree	273	14.1
Disagree or strongly disagree	7	0.4
I believe the booster dose will be useful in protecting me from COVID-19 infection		
Agree or strongly agree	1699	87.5
Neither agree nor disagree	238	12.3
Disagree or strongly disagree	5	0.3
I believe the benefits of the COVID-19 vaccine outweigh the risks		
Agree or strongly agree	1672	86.1
Neither agree nor disagree	264	13.6
Disagree or strongly disagree	6	0.3
I believe if I get vaccinated, the risk of contracting COVID-19 or infecting others will be reduced		
Agree or strongly agree	1735	89.3
Neither agree nor disagree	189	9.7
Disagree or strongly disagree	18	0.9
I am worried about the halal status of the new booster dose of COVID-19 vaccine		
Agree or strongly agree	581	29.9
Neither agree nor disagree	744	38.3
Disagree or strongly disagree	617	31.8
Getting a booster dose vaccinated takes a lot of time and effort		
Agree or strongly agree	656	33.8
Neither agree nor disagree	697	35.9
Disagree or strongly disagree	589	30.3

**Table 4:** Final multivariable linear regression model showing factors associated with the willingness-to-pay for a booster dose COVID-19 vaccine in Indonesia (*n* = 1942).

Variable	Unstandardized Coefficients				*p* Value	US$ Estimate		
	β	95% CI ^3^ of β		SE		Mean	95% CI	
		Lower	Upper				Lower	Upper
Gender (*Male*)								
Female	−0.082	−0.195	0.032	0.058	0.158	1.723	−0.471	3.917
Age (*31–40*)								
≤20	0.096	−0.134	0.325	0.117	0.415	2.058	−0.136	4.252
21–30	0.120	−0.026	0.267	0.075	0.107	2.109	−0.085	4.303
41–50	−0.236	−0.459	−0.012	0.114	0.039	1.477	−0.717	3.671
>50	−0.147	−0.397	0.104	0.128	0.251	1.615	−0.579	3.809
Higher education (*Elementary/high school*)								
Diploma	0.050	-0.112	0.211	0.082	0.546	1.965	−0.228	4.159
Undergraduate/graduated	0.339	0.105	0.572	0.119	0.004	2.624	0.430	4.818
Religion (*Islam*)								
Christian (Protestant)	0.067	−0.118	0.251	0.094	0.479	1.999	−0.195	4.193
Catholic	0.191	−0.020	0.402	0.107	0.076	2.264	0.070	4.457
Other (Hindu/Buddha/Atheist or agnostic/Confucianism)	0.284	0.079	0.489	0.105	0.007	2.485	0.291	4.679
Occupation (*Homemaker/Self-employed*)								
Employed for wages	0.314	0.055	0.573	0.132	0.018	2.559	0.366	4.753
Student + Retired/unable to work/others	0.337	0.071	0.603	0.136	0.013	2.620	0.426	4.814
Monthly household income (IDR ^1^) *(<3 million*)								
3–5 million	−0.004	−0.181	0.174	0.091	0.966	1.863	−0.331	4.057
5–10 million	0.136	−0.035	0.308	0.088	0.120	2.143	−0.051	4.337
>10 million	0.481	0.274	0.689	0.106	<0.001	3.026	0.833	5.220
Having family member seriously ill or died caused by COVID-19? (*No*)								
Yes	0.076	-0.040	0.192	0.059	0.198	2.018	−0.176	4.212
Having influenza vaccinated for the last 5 years? (*No*)								
Yes	0.220	0.087	0.352	0.067	0.001	2.329	0.136	4.523
Booster dose can provide immune better after the second dose (*Yes*)								
No/I do not know	−0.295	−0.485	-0.106	0.097	0.002	1.392	−0.802	3.585
Booster dose can stimulate antibody production to fight COVID-19 infection (*Yes*)								
No/Not sure	0.196	−0.060	0.453	0.131	0.133	2.276	0.082	4.470
Booster dose can lower hospitalization rate if infected by COVID-19 (*Yes*)								
No/Not sure	−0.005	−0.210	0.200	0.105	0.961	1.861	−0.333	4.054
Booster dose can protect the unvaccinated people (*Yes*)								
No/Not sure	−0.175	−0.305	−0.045	0.066	0.008	1.570	−0.624	3.764
The booster dose is important to protect the public from COVID-19 (*No/I do not know*)								
Yes	0.057	-0.200	0.315	0.131	0.663	1.980	−0.213	4.174
I believe that natural immunity is sufficient and I do not need to be vaccinated (*Disagree or strongly disagree*)								
Agree or strongly agree	0.013	−0.216	0.242	0.117	0.914	1.894	−0.300	4.088
Neither agree nor disagree	−0.256	−0.419	−0.093	0.083	0.002	1.448	−0.746	3.642
COVID-19 infection is harmless, so I do not have to be vaccinated (*Disagree or strongly disagree*)								
Agree or strongly agree	0.294	0.026	0.561	0.136	0.031	2.509	0.315	4.703
Neither agree nor disagree	−0.209	−0.451	0.032	0.123	0.089	1.517	−0.677	3.711
I am not sure vaccination is effective against COVID-19 (*Disagree or strongly disagree*)								
Agree or strongly agree	0.110	−0.086	0.305	0.100	0.271	2.087	−0.107	4.281
Neither agree nor disagree	−0.022	−0.187	0.143	0.084	0.797	1.830	−0.364	4.024
I am worried about any adverse side effects or allergic reactions when vaccinated with booster doses (*Disagree or strongly disagree*)								
Agree or strongly agree	−0.026	−0.203	0.151	0.090	0.769	1.821	−0.373	4.015
Neither agree nor disagree	−0.022	−0.193	0.148	0.087	0.798	1.829	−0.365	4.023
Complications may arise after receiving the booster dose (*Disagree or strongly disagree*)								
Agree or strongly agree	0.072	−0.128	0.272	0.102	0.48	2.010	−0.184	4.203
Neither agree nor disagree	−0.127	−0.253	−0.001	0.064	0.049	1.647	−0.546	3.841
I am worried about the unexpected effect of booster dose in the future (*Disagree or strongly disagree*)								
Agree or strongly agree	−0.152	−0.325	0.021	0.088	0.085	1.606	−0.587	3.8
Neither agree nor disagree	−0.135	−0.288	0.017	0.078	0.082	1.633	−0.561	3.827
I believe if I get vaccinated, the risk of contracting COVID-19 or infecting others will be reduced (*Disagree or strongly disagree*)								
Agree or strongly agree	0.603	0.075	1.131	0.269	0.025	3.418	1.224	5.612
Neither agree nor disagree	0.669	0.109	1.228	0.285	0.019	3.650	1.456	5.844
I am worried about the halal status of the new booster dose of COVID-19 vaccine (*Disagree or strongly disagree*)								
Agree or strongly agree	−0.185	−0.344	−0.026	0.081	0.023	1.554	−0.639	3.748
Neither agree nor disagree	0.002	−0.136	0.139	0.070	0.981	1.873	−0.321	4.067
Getting a booster dose vaccinated takes a lot of time and effort (*Disagree or strongly disagree*)								
Agree or strongly agree	0.031	−0.112	0.174	0.073	0.667	1.930	−0.264	4.124
Neither agree nor disagree	0.021	−0.114	0.156	0.069	0.762	1.910	−0.284	4.103
MSE ^2^	1.252							
F-value (*p* < 0.001)	6.190							
R^2^	0.115							

^1^ IDR: Indonesian Rupiah; ^2^ MSE: Mean squared error; ^3^ CI: Confidence interval. The IDR-to-US$ exchange rate is IDR 14,835/US$ (31 August 2022 currency rate).

## Data Availability

The data supporting the results of this study are available upon request through contacting the first corresponding author (H.H.).
